# Two new apterous species of *Lathrobium* Gravenhorst (Coleoptera, Staphylinidae, Paederinae) from Fujian, East China

**DOI:** 10.3897/zookeys.218.3361

**Published:** 2012-08-30

**Authors:** Zhong Peng, Li-Zhen Li, Mei-Jun Zhao

**Affiliations:** 1 Department of Biology, College of Life and Environmental Sciences, Shanghai Normal University, Shanghai, 200234, P. R. China

**Keywords:** Coleoptera, Staphylinidae, taxonomy, *Lathrobium*, new species, Fujian, China

## Abstract

Two new apterous species of the genus *Lathrobium* Gravenhorst, 1802 from Fujian Province, East China, *Lathrobium daocongchaoi* Peng & Li, **sp. n.** and *Lathrobium fujianense* Peng & Li,**sp. n.**, are described and illustrated.

## Introduction

So far, 62 species of the genus *Lathrobium* Gravenhorst, 1802 have been reported from China (Peng et al. 2012c), but none was previously known from Fujian Province, East China. Only eleven species have been reported from the adjacent Zhejiang Province (Watanabe and Luo 1992, 1999a, b; Peng et al. 2012a, b).

Recently, the first author and his colleagues made two collecting trips to Fujian Province and collected a small series of *Lathrobium*. The examination of the specimens revealed two species, both of which are remarkably different from the previously known species from China regarding their male sexual characters.

## Material and methods

All specimens were collected from the leaf litter in broad-leaved forests by sifting. The following abbreviations are used in the text, with all measurements in millimeters:

BL length of body from the anterior margin of the labrum to the apex of the abdomen;

FL length of forebody from the anterior margin of the clypeus to the posterior margin of the elytra;

HL length of head from the anterior margin of the clypeus to the posterior margin of the head;

HW maximum width of head;

PL length of pronotum along midline;

PW maximum width of pronotum;

EL length of elytra from the apex of the scutellum to the posterior margin of the elytra.

The type material is deposited in the Insect Collection of Shanghai Normal University, Shanghai, China. (**SNUC**).

### Descriptions

#### Lathrobium daicongchaoi

Peng & Li sp. n.

urn:lsid:zoobank.org:act:8FE90F67-320E-49F1-A324-67DEC71B6D5F

http://species-id.net/wiki/Lathrobium_daicongchaoi

Figs 1A , 2

##### Type material

(3 ♂♂, 6 ♀♀)**.** Holotype: ♂, labeled ‘**CHINA:** Fujian Prov. / Wuyishan City / Guadun Village / 27°44'N, 117°37'E / 26.v.2012, alt. 1,400 m / Dai & Peng leg.’. Paratypes: 2♀♀, same label data as holotype; 2 ♂♂, 3 ♀♀, same data, except ‘29.v.2012’; 1♀, same data, except ‘28.v.2012’.

##### Description.

Measurements and ratios:BL 7.51–8.90, FL 3.72–4.00, HL 0.89–1.02, HW 1.02–1.15, PL 1.33–1.52, PW 1.13–1.24, EL 0.93–0.96, HL/HW 0.86–0.92, HW/PW 0.90–0.94, HL/PL 0.65–0.67, PL/PW 1.18–1.24, EL/PL 0.63–0.70.

**Figure 1.**
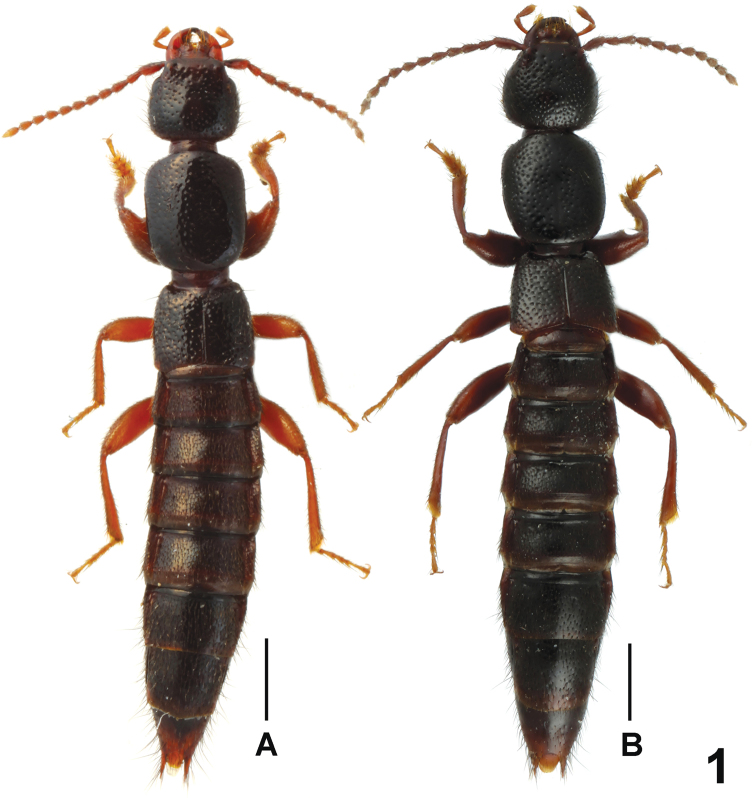
Male habitus of *Lathrobium* spp., **A** *Lathrobium daicongchaoi* **B** *Lathrobium fujianense*. Scales: 1.0 mm.

Habitus as in Fig. 1A. Body brown with paler apex, legs light brown, antennae light brown to yellowish brown.

Head subquadrate (HL/HW 0.86–0.92); punctation coarse and sparse; interstices with very shallow microreticulation; eyes small, approximately 1/3–2/5 the length of postocular region in dorsal view.

Pronotum nearly parallel-sided; punctation sparser than that of head; impunctate midline narrow; interstices shining without microsculpture.

Elytra with punctation denser than that of pronotum and well defined; hind wings reduced.

Abdomen with dense punctation; interstices with very shallow, transversely striate microsculpture.

**Figure 2.**
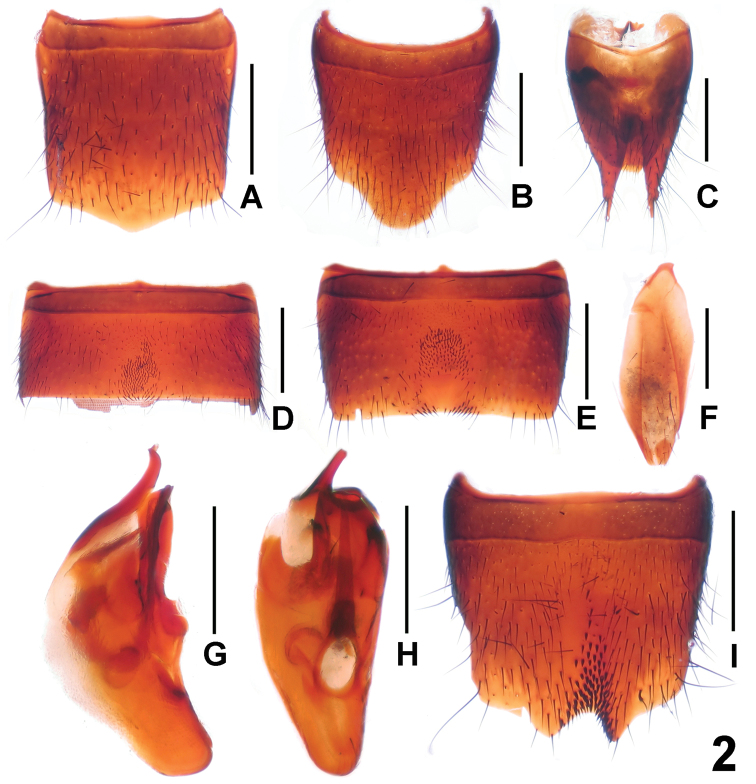
*Lathrobium daicongchaoi*. **A** female tergite VIII **B** female sternite VIII **C** female tergites IX–X **D** male sternite VI **E** male sternite VII **F** male sternite IX**G**﻿ aedeagus in lateral view **H** aedeagus in ventral view **I** male sternite VIII. Scales: 0.5 mm.

Male. Sternite VI (Fig. 2D) with dense darkish setae in postero-median impression; sternite VII (Fig. 2E) with sparse darkish setae in median impression, and posterior margin with several peg-like setae; sternite VIII (Fig. 2I) with asymmetric emargination and short darkish setae in shallow impression; sternite IX (Fig. 2F) nearly symmetric; aedeagus (Figs 2G, 2H) with short ventral process and thin dorsal sclerite.

Female. Posterior margin of tergite VIII (Fig. 2A) pointed in middle; sternite VIII (Fig. 2B) slightly longer than that of male, middle of posterior margin with broad and obtuse projection; tergite X (Fig. 2C) obtuse apically and not reaching anterior margin of tergite IX (Fig. 2C).

##### Distribution.

East China: Fujian.

##### Etymology.

The species is named after Cong-Chao Dai, collector of the type specimens.

##### Remarks.

The new species resembles *Lathrobium zhaotiexiongi* Peng & Li, 2012 in havinga postero-median impression on the male sternite VI and similarly shaped male sternite IX. The new species can be readily separated by the presence of a median impression on the male sternite VII and by the short ventral process of the aedeagus. In *Lathrobium zhaotiexiongi*, the male sternite VII lacks a median impression and has an asymmetric posterior emargination , and the ventral process of the aedeagus is longer.

#### Lathrobium fujianense

Peng & Li sp. n.

urn:lsid:zoobank.org:act:7979EA9D-2FC1-4E70-AE5F-2A794BABF36F

http://species-id.net/wiki/Lathrobium_fujianense

Figs 1B , 3

##### Type material

(1 ♂, 2 ♀♀)**.** Holotype: ♂, labeled ‘**CHINA:** Fujian Prov. / Mingxi County / Mt. Junzifeng / 26°34'N, 117°16'E / 7.viii.2008, alt. 1,400 m / Qi & Yin leg.’. Paratypes: 2 ♀♀, same label data as holotype.

##### Description.

Measurements and ratios:BL 7.78–9.73, FL 3.77–4.05, HL 1.11–1.13, HW 1.24–1.26, PL 1.48–1.54, PW 1.30–1.33, EL 0.93–0.96, HL/HW 0.90, HW/PW 0.95, HL/PL 0.73–0.75, PL/PW 1.14–1.16, EL/PL 0.62–0.63.

Habitus as in Fig. 1B. General appearance similar to that of *Lathrobium daicongchaoi*, except for the darker coloration, somewhat larger body size, denser punctation on head and pronotum, and the weakly convex lateral margins of pronotum in dorsal view.

**Figure 3.**
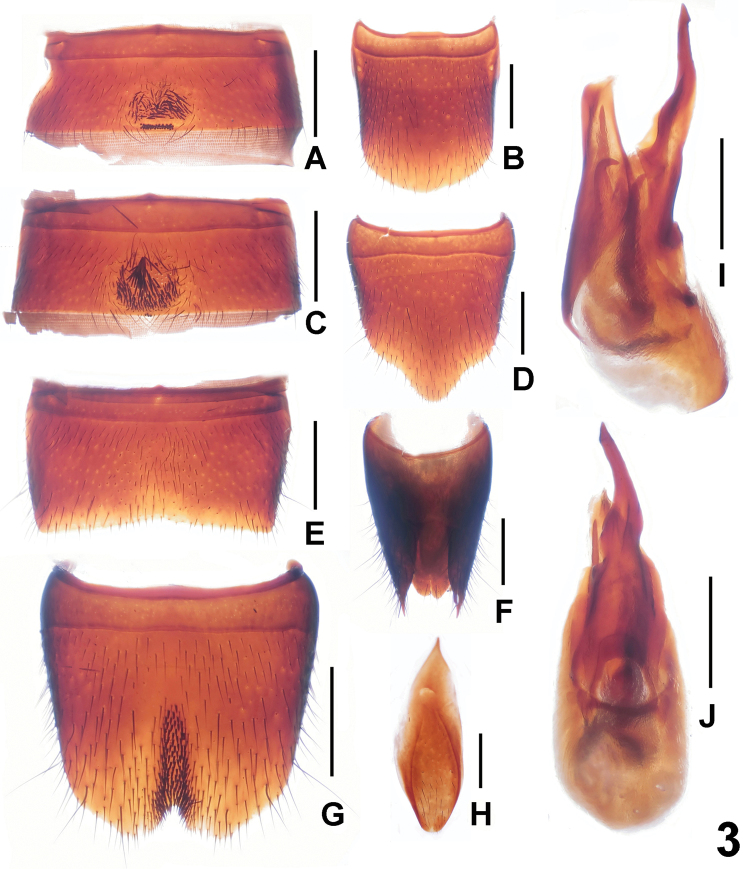
*Lathrobium fujianense*. **A** male sternite V **B** female tergite VIII **C** male sternite VI **D** female sternite VIII **E** male sternite VII **F** female tergites IX–X**G** male sternite VIII **H** male sternite IX **I** aedeagus in lateral view **J** aedeagus in ventral view. Scales: 0.5 mm.

Male. Sternite V (Fig. 3A) with darkish setae in postero-median impression, and posterior margin with several point-like setae; sternite VI (Fig. 3C) similar to sternite V, but with much fewer point-like setae; posterior margin of sternite VII (Fig. 3E) weakly concave; sternite VIII (Fig. 3G) with subtriangular, weakly asymmetric emargination and short darkish setae in the narrow median impression; sternite IX (Fig. 3H) nearly symmetric; aedeagus (Fig. 3I, 3J) with long ventral process and short dorsal sclerite.

Female. Posterior margin of tergite VIII (Fig. 3B) broadly convex; sternite VIII (Fig. 3D) longer than that of male, middle of apical margin with obtuse projection; tergite X (Fig. 3F) broadly convex apically and not reaching anterior margin of tergite IX (Fig. 3F).

##### Distribution.

East China: Fujian.

##### Etymology.

The specific epithet is derived from Fujian Province, where the type locality is situated.

##### Remarks.

The new species can be separated from other East Chinese *Lathrobium* species by the morphology of the aedeagus, as well as by the presence of point-like setae at the posterior margin of the male sternite V and by the presence of short dark setae in the narrow postero-median impression of the male sternite VIII.

## Supplementary Material

XML Treatment for Lathrobium daicongchaoi

XML Treatment for Lathrobium fujianense
